# Systematic Review of Clinical Trials Assessing the Effectiveness of Ivy Leaf (Hedera Helix) for Acute Upper Respiratory Tract Infections

**DOI:** 10.1155/2011/382789

**Published:** 2010-10-03

**Authors:** Felix Holzinger, Jean-François Chenot

**Affiliations:** Department of General Practice/Family Medicine, University Medicine Goettingen, Humboldtallee 38, 37073 Goettingen, Germany

## Abstract

*Introduction*. Among nonantibiotic cough remedies, herbal preparations containing extracts from leaves of ivy (Hedera helix) enjoy great popularity. *Objective*. A systematic review to assess the effectiveness and tolerability of ivy for acute upper respiratory tract infections (URTIs). *Methods*. We searched for randomized controlled trials (RCTs), nonrandomized controlled clinical trials and observational studies evaluating the efficacy of ivy preparations for acute URTIs. Study quality was assessed by the Jadad score or the EPHPP tool. *Results*. 10 eligible studies were identified reporting on 17463 subjects. Studies were heterogeneous in design and conduct; 2 were RCTs. Three studies evaluated a combination of ivy and thyme, 7 studies investigated monopreparations of ivy. Only one RCT (*n* = 360) investigating an ivy/thyme combination used a placebo control and showed statistically significant superiority in reducing the frequency and duration of cough. All other studies lack a placebo control and show serious methodological flaws. They all conclude that ivy extracts are effective for reducing symptoms of URTI. *Conclusion*. Although all studies report that ivy extracts are effective to reduce symptoms of URTI, there is no convincing evidence due to serious methodological flaws and lack of placebo controls. The combination of ivy and thyme might be more effective but needs confirmation.

## 1. Introduction

Cough is a highly prevalent condition and a common reason for consultations in general practice [1–4]. Most frequently, cough symptoms are caused by acute viral upper respiratory tract infections (URTIs) and the course is mostly benign and self-limiting, although bacterial superinfection may occur in acute bronchitis [5, 6]. For chronic cough, important causes are chronic obstructive pulmonary disease (COPD) and asthma which are characterized by airway obstruction and hypersecretion of mucus, additionally causing symptoms like wheezing or dyspnoea. 

Inappropriate use of antibiotics for viral respiratory tract infections is a significant problem causing both pathogen resistance and substantial health care expenditure without affecting the resolution of cough [7]. Therefore nonantibiotic alternative treatment options are needed. Commonly used over-the-counter drugs for acute cough in both children and adults are mucolytic agents and antitussives, which are also widely prescribed in primary care settings [8]. In the UK, cough liquids accounted for sales worth 102 million pounds in 2008 [9]. Among these nonantibiotic cough remedies, herbal preparations containing extracts from the leaves of ivy (Hedera helix L.) enjoy great popularity in many European countries [10–12]. In 2007, more than 80% of herbal expectorants prescribed in Germany comprised ivy extract and amounted to nearly 2 million prescriptions nationwide and a volume of sales exceeding 13 million Euros [10]. 

 Ivy leaf contains saponins which are considered to have mucolytic, spasmolytic, bronchodilatory and antibacterial effects [13, 14]. Despite widespread use of ivy leaf extracts, the effectiveness for the treatment of acute cough is not well established. Methodically strong clinical studies seem scarce despite the epidemiological and economic importance. To our knowledge, there is no comprehensive systematic review of the available clinical evidence. A Cochrane Review assessing over-the-counter cough medications does not cover herbal drugs [8]. Other reviews focus on effectiveness of ivy leaf extracts in asthma or COPD [12, 15–17]. Therefore, we performed a systematic review of the effectiveness and tolerability of ivy preparations for the treatment of acute URTIs in children and adults.

## 2. Methods

### 2.1. Data Sources

Our search included 3 electronic bibliographic databases: MEDLINE, EMBASE and the Cochrane Library. We included studies published from the respective inception of the databases until 20. December 2009. There was no language restriction. Search terms were: ivy, hedera, respiratory tract diseases, respirat*, cough, bronchitis, bronchial. The complete search algorithm with the keywords and MeSH-terms used is available from the authors upon request. Additionally, we hand searched the bibliographies of the publications retrieved. Several manufacturer websites were also scanned manually for references.

### 2.2. Study Selection

#### 2.2.1. Eligibility Criteria

We did not exclude specific populations or age groups. Our search included published randomized controlled trials (RCTs), controlled clinical trials (CCTs) and noncontrolled observational studies (OSs) evaluating the effectiveness and/or tolerability of medications containing ivy leaf extract for the treatment of acute URTIs including bronchitis. Studies investigating patients with a variety of other acute (e.g., pertussis, pneumonia) and chronic diseases (COPD, asthma) were excluded. However, we did not disregard studies where URTI patients represented the majority of investigated subjects or treatment results were reported separately. Ivy leaf extract could be the only ingredient in the respective drug preparation or could be combined with other herbal components. We restricted the search on studies evaluating oral or rectal administration forms. Outcome measures could be hard clinical endpoints (e.g., morbidity, mortality, health-related quality of life), surrogate values like spirometric parameters, physician's findings upon clinical examination, assessment of symptoms (e.g., cough) by either physician or patient, and undesirable side effects of treatment.

#### 2.2.2. Screening Process

**Figure 1:**
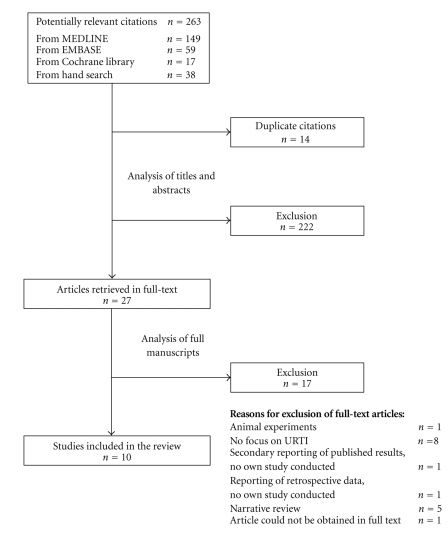
Study selection process.

The titles and abstracts of the citations identified were screened by two independent reviewers (FH and JFC) separately using a predesigned form. Titles and abstracts that clearly did not meet the inclusion criteria regarding indication (respiratory symptoms) or intervention (drug containing ivy leaf extract) were excluded. Duplicate titles were also eliminated. For publications fulfilling the inclusion criteria or for which inclusion or exclusion could not be ascertained, we reviewed the full text. Disagreements were resolved by consensus. A list of references of excluded studies is available upon request from the authors.  Figure 1 shows a detailed outline of the study selection process.

### 2.3. Quality Assessment and Data Extraction

For quality assessment of RCT, the Jadad scale (score 0–5) was used [18]. The Jadad scale is not designed for assessment of nonrandomized or noncontrolled studies as it covers mainly the study characteristics of blinding and attrition [18, 19]. To assess the methodological quality of CCTs and OSs we used the Quality Assessment Tool for Quantitative Studies developed by the Effective Public Health Practice Project (EPHPP, McMaster University, Ontario, Canada) [20, 21]. Based on several component ratings (selection bias, study design, confounders, blinding, data collection methods, withdrawals), studies are given a global rating of either “strong”, “moderate” or “weak”. RCTs were given both a Jadad score and an EPHPP rating. Data was then extracted using a predesigned spreadsheet. These steps of critical quality appraisal and data extraction were performed independently by the two reviewers. Disagreements were resolved by consensus.

### 2.4. Data Analysis

The included studies were categorized according to their study design: RCT, CCT or OS. For controlled studies, the following comparisons were made: (1) ivy leaf extract versus placebo, (2) ivy leaf extract versus conventional therapy, (3) comparison of different formulations of Ivy leaf extract. For OSs findings before and after treatment are reported. Due to highly heterogeneous outcomes used by the included studies, we did not attempt to calculate pooled results.

## 3. Results

### 3.1. Characteristics of Included Studies

Our search identified 263 potentially relevant citations. Of these, 27 publications were retrieved for evaluation of the full text. We retained 10 studies for inclusion into the review. The studies were published between 1985 and 2009 and originated from a variety of countries (Germany *n* = 5, Switzerland *n* = 2, Latin America *n* = 2, Ukraine *n* = 1). The studies report on a total of 17463 subjects in treatment and control groups. Three of these studies included only children, 2 studies only adults and 5 studies included both. Studies were heterogeneous in design and conduct, 2 were RCT, one was a CCT and 7 were OS. Of the 3 controlled studies, only one was placebo-controlled [22], one compared ivy leaf to a conventional expectorant (acetylcysteine) [23]. The third study compared two different syrup formulations containing ivy leaf extract [24]. Most studies investigated mono-preparations of ivy leaf extract, but 3 studies tested a mixture of ivy leaf and thyme extract [22, 25, 26]. One of these used a randomized controlled design [22].

The studies included patients with cough due to URTIs including acute bronchitis. Some studies included few patients with chronic bronchitis [26, 27], or the authors did not differentiate distinctly between acute disease and acute exacerbations of chronic disease [27, 28]. One study included COPD and pertussis patients, but reported the results separately [29]. The outcomes assessed by the studies were heterogeneous. Studies reported assessment of URTI symptoms by the treating physician or results of physical examination. Symptoms assessed varied (e.g., urge to cough, frequency of coughing, quality and quantity of sputum, shortness of breath, auscultation results). Symptom severity was measured by a variety of instruments. Two studies [22, 26] used the BSS (bronchitis severity score) scale. Several studies mainly reported percentages of improvement or cure of symptoms after a certain treatment period. Two studies additionally reported a global self-assessment by patients [25, 30]. One study measured and reported spirometric parameters [23]. An overview of the characteristics of the studies is given in Table 1. 

Only one RCT met the standards of good clinical practice with regard to conduct and reporting, sample size calculation and an appropriate statistical analysis and was rated with a Jadad score of 5 (“strong”) [22]. All other studies, including the second RCT [24] had serious flaws. It was often impossible to discern whether the participants were representative of the target population of unselected URTI patients. The studies did not describe the selection process or relied on self-selected participants (selection bias). Confounding was a problem in a large number of studies as well. Many studies allowed concomitant use of other cough remedies or even antibiotics or steroids [23, 29, 30] or did not address this factor at all [26]. Presence of concomitant diseases (e.g., cardiovascular) was not an exclusion criterion [23, 25, 29] or not assessed [26, 30]. Dropout rates or reasons for dropout were frequently not reported [27–31]. No intention-to-treat analysis was reported. Statistical analysis was performed by only few studies [22–24, 27], most studies just describe the results. One study reporting *P*-values does not mention the statistical test performed [23].  Table 2 shows details of the quality assessment. 

 The reported results are shown in Table 3 and summarized in the following sections.

### 3.2. Ivy Leaf Extract versus Placebo

No studies comparing ivy leaf mono-preparations for the treatment of URTIs to a placebo control could be identified. One German RCT compared ivy/thyme to placebo [22]. This trial included 370 adult patients which were treated for 10 days with a syrup (ivy 1.5 g liquid extract/100 g 1 : 1, ethanol 70%, thyme 15 g liquid extract/100 g 1: 2–2.5). Outcome measures were the frequency of coughing fits, the time to a relative 50% reduction in coughing fits, the BSS and overall response rate. All outcomes assessed were significantly better in the intervention group.

### 3.3. Ivy Leaf Extract versus Conventional Expectorant Therapy

One Ukrainian study group conducted a CCT comparing ivy to acetylcysteine in acute bronchitis [23]. A total of 50 children were treated for 7–10 days with either syrup containing ivy leaf extract or 300–1200 mg of acetylcysteine. Symptoms (cough, sputum, shortness of breath) were improved in both groups after treatment. Compared to the baseline, both treatment groups showed improved spirometric values (FVC, FEV1, PEF) after treatment. Spirometric parameters were significantly better in the group receiving the ivy formulation. The authors conclude that ivy leaf has bronchodilatory as well as mucolytic effects.

### 3.4. Comparison of Different Formulations of Ivy

One of the two RCTs included in this systematic review compared two different syrup preparations of ivy leaf extract (0.33–0.5 g dry extract/100 ml 3–6 : 1, ethanol 60%, versus 0,7 g dry extract/100 ml 5–7.5 : 1, ethanol 30%) [24]. A total of 52 patients were treated for 10 days after randomization, severity of cough symptoms was assessed. No significant difference was observed.

### 3.5. Observational Studies

Five noncontrolled OSs investigated ivy mono-preparations [27, 31] and 2 OSs investigated ivy/thyme combinations [25, 26]. These studies included a large number of patients, the 2 largest studies (both conducted in Latin America) [27, 31] together reporting on over 15000 subjects. Most of the OSs were postmarketing surveillance-studies sponsored by the manufacturers. Symptoms were assessed at baseline and after treatment for 7 to 10 days, mostly by the investigating physicians. Different symptoms (e.g., cough, expectoration, shortness of breath) were assessed on a variety of scales. Two studies used a four-level Likert scale [28, 30]. Most studies observed improvement or cure after treatment in more than 90%. Global efficacy was rated as good or very good by physicians in 77–86% [25, 28, 30]. One study assessing auscultation findings reported significantly less rales after treatment compared to baseline [27].

### 3.6. Adverse Events

All but one publication studied adverse events as an outcome parameter [24]. Side effects were observed in 0% [30] to 3.8% [22]. Symptoms were mostly mild and transient abdominal discomfort, diarrhoea or nausea. One study reported allergic reactions in 0.1% of patients (10 cases in a large study population of over 10000 patients) [31]. In another study one patient out of 248 (0.4%) who was concomitantly treated with paracetamol developed an allergic skin rash [28]. Severe or fatal adverse events were not reported. In the RCT comparing an ivy/thyme preparation to placebo [22], rates for frequency of observed side effects were similar in the treatment (3.8%) and placebo (4.5%) group.

## 4. Discussion

### 4.1. Effectiveness

All studies reviewed concluded that ivy leaf extract is effective for the treatment of URTIs. After 7 to 10 days of treatment, symptoms like cough or expectoration are improved or cured in a large majority of patients. It has to be noted that only one placebo-controlled RCT could be retrieved. This trial showed superior results as to symptom relief and speed of recovery in the intervention group treated with a combination drug of ivy and thyme extract [22].

 For ivy mono-preparations, sound evidence remains scarce. All studies investigating a mono-preparation of ivy extract lacked a placebo control. Only one study with severe methodological flaws compared ivy to acetylcysteine [23]. The remaining observational studies all observed an improvement of URTI symptoms in the course of treatment. Although the authors of these studies claim to have established effectiveness, this conclusions are not warranted since the natural course of URTIs is usually benign and self-limiting. Uncomplicated acute bronchitis usually presents with a phase of variable constitutional symptoms that lasts for one to five days, followed by cough and phlegm production lasting for one to 3 weeks, whereas cough lasting for more than 21 days can be defined as subacute or chronic and requires further diagnostic workup [32]. A review of the regression of symptoms in children with acute cough observed that after 5 to 8 days, symptoms were improved or gone in 50–75% of patients or patients were assessed as recovered by their physicians [33]. After one week, cough was resolved in about 50%. Improvement rates shown in the Hedera helix studies included in this review tend to be higher, often exceeding 90% [28, 29, 31]. However, these recovery rates can not serve as direct comparison because many other confounders (comedication, comorbidity) affecting the outcome have to be considered. Therefore, studies using placebo controls are indispensable to establish effectiveness. 

 Only for the combination of ivy and thyme evidence for effectiveness of faster recovery could be found [22]. A 50% reduction in coughing fits was reached 2 days earlier in the treatment group, which can be considered a clinically relevant improvement. It remains unclear if the observed effect can be attributed to the action of thyme or the combination. Although thyme is traditionally used for URTIs, we found no clinical trials or a systematic review evaluating the effectiveness of mono-preparations of thyme. Blinding might have been a problem since producing placebos with comparable taste and smell for oral herbal therapies is difficult.

### 4.2. Tolerability

The benefits of any treatment have to be weighted against potential harms. As severe adverse events were not reported with more than 17000 subjects treated, there is considerable evidence for the safety of *hedera helix*. In the placebo-controlled study, only minimal differences in adverse events between verum and placebo group were observed [22]. Allergic reactions were reported in under 0.5% of cases with no cases of severe anaphylaxis mentioned in any study. This is especially important regarding the fact that *hedera helix* has been reported to cause allergic contact dermatitis [34, 35] and rarely even occupational asthma in gardeners exposed to the leaves [36].

### 4.3. Strengths and Limitations

We comprehensively searched medical databases with no restrictions regarding time or language. An extensive hand-searching process added further studies for review, so we can be confident as to the completeness of the evidence identified. However, several limitations have to be taken into account. Firstly, the databases covered mainly focus on the American and European literature. As *hedera helix* products are also marketed in the Middle East and East Asia, some studies published in local journals and not listed in MEDLINE or EMBASE could have been missed. Furthermore, a large number of included studies was sponsored by the manufacturers [22, 25–29, 31] or funding was not reported. Studies sponsored by the manufacturer are prone to publication bias [37]. One study was published in a section of a phytotherapy journal in which articles originating from pharmaceutical companies could be published without any editing or review [29]. 

 A quantitative synthesis of the data gathered from the studies (e.g., meta-analysis) was not possible due to low-quality data and great heterogeneity in study designs and outcomes. Future trials will require a consensus on a standard for measuring symptom relief in URTIs.

## 5. Conclusion

Although many studies conclude that ivy extracts are effective to reduce symptoms of acute URTIs, their effectiveness is not established beyond reasonable doubt. The studies reviewed show serious methodological flaws and lack placebo controls. Therefore their findings must be interpreted with due caution. For a combination of ivy and thyme, effectiveness has been demonstrated in one RCT, however these findings need to be confirmed. Ivy extracts are well tolerated, no serious adverse events were reported. It has to be taken into account that other popular cough medications, for example, acetylcysteine [38] and other OTC antitussives and decongestants [8], also lack a sound evidence base. Reducing the use of antibiotics for uncomplicated URTIs is an important public health goal [39]. Given a high desire for treatment, abandoning the use of non-evidence-based cough remedies might cause a surge in the prescription of antibiotics, which poses the risks of serious adverse events and the development of antibiotic resistance. Considering the popularity of ivy preparations and the considerable expenditure for such remedies, further rigorously designed randomized controlled trials are necessary.

##  Conflict of Interest Statement 

The authors declare that they have no proprietary, financial, professional or other personal interest of any nature or kind in any product, service and/or company that could be construed as influencing the position presented in this paper.

##  Funding 

This work was not supported by any external funding.

##  Contribution of the Authors 

The literature search was done by F. Holzinger, selection and appraisal of studies and writing of the manuscript was done by F. Holzinger and J. F. Chenot.

## Figures and Tables

**Table 1:** Characteristics of included studies.

Reference	Country	Patients (I/C)*	Inclusion criteria	C/A**	Gender (m/f in %)	Age range (years)	Treatment				Treatment (days)
							Intervention group	daily dose	Control group	daily dose	
Randomized controlled trials (RCTs)											

Kemmerich et al. 2006	Germany	182/178	Acute bronchitis	A	46.7/53.3	18–87 y	Ivy + Thyme, “Bronchipret” syrup Ivy: 1.5 g LE/100 g (1:1, E70%) Thyme: 15 g LE/100 g (1:2–2.5)	16.2 ml	Placebo	n.a.	10
Unkauf and Friedrich 2000	Germany	25/27	Acute bronchitis	C	51.9/48.1	<12 y	Ivy, “Valverde” syrup 0.33–0.5 g DE/100 ml (3–6:1, E60%)	10–20 ml	Ivy, “Prospan” syrup 0.7 g DE/100 ml (5–7.5:1, E30%)	10–20 ml	10

Controlled trials (CCTs)											

Bolbot et al. 2004	Ukraine	25/25	Acute bronchitis	C	64.0/36.0	2–10 y	Ivy, “Prospan” syrup (5–7.5:1)	15–30 ml	Acetylcysteine	300–1200 mg	7–10

Observational studies (OSs)											

Büechi and Kähler 2003	Switzerland	56	Acute bronchitis, common cold	C/A	32.1/67.9	7–93 y	Ivy, pastilles 26 mg DE/pastille (4–8:1, E30%)	2–6 pastilles, 52–156 mg DE			7
Büechi et al. 2005	Switzerland	62	Acute and chronic bronchitis, common cold	A	40.3/59.7	16–89 y	Ivy + Thyme, syrup, Ivy: 1.0 g DE/100 g (4–8:1, E30%) Thyme: 25.4 g DE/100 g (1:3.5–4)	5–15 ml (6.7–20.1g)			3–23
Fazio et al. 2009	Latin America***	9657	Acute and chronic bronchitis, cough	C/A	NR	0–98 y	Ivy, syrup (5–7.5:1, E30%)	7.5–22.5 ml			7
Hecker 1999	Germany	248	Bronchitis	C/A	44.4/55.6	0–70 y	Ivy, “Prospan” syrup in 48.4% of patients, “Prospan” effervescent tablets in 51.6% of patients	NR	n.a.		~8 (mean)
Leskow 1985	Germany	84	Acute and chronic bronchitis, chesty cough, pertussis	C/A	46.4/53.6	1–82 y	Ivy, “Prospan” drops 2 g DE/100 ml	30–100 drops			12.3 (mean)
Marzian 2007	Germany	1044	Acute bronchitis	C	50.5/49.5	0–17 y	Ivy + Thyme, “Bronchipret” syrup Ivy: 1.5 g LE/100 g (1:1, E70%) Thyme: 15 g LE/100 g (1:2–2.5)	9.6–16.2 ml			10
Santoro Júnior 2005	Brasil	5850	Bronchitis	C/A	47.9/52.1	NR	Ivy, “Abrilar” syrup	7.5–22.5 ml			7

n.a. = not applicable, NR = not reported, DE = dry extract, LE = liquid extract, E% = ethanol %, Values are reported as stated in the respective publications. If information is missing (e.g., composition of herbal preparation), the paper does not give any more details. Brand names are marked by quotation marks. *Number of participants included in analysis (I/C = number in Intervention/Control group) **C/A = Children/Adults, ***11 countries in Latin America.

**Table 2:** Quality assessment of included studies.

Reference	EPHPP Section ratings (strong/moderate/weak)						Global rating	Jadad score(0–5)
	Selection bias	Study design	Confounders	Blinding	Data collection	Withdrawals		
Randomized controlled trials (RCTs)								

Kemmerich et al. 2006	moderate	strong	strong	strong	weak	strong	strong	5
Unkauf and Friedrich 2000	weak	strong	moderate	weak	weak	strong	weak	2

Controlled trials (CCTs)								

Bolbot et al. 2004	moderate	moderate	weak	weak	moderate	strong	weak	n.a.

Observational studies (OSs)								

Büechi and Kähler 2003	weak	moderate	weak	weak	weak	weak	weak	
Büechi et al. 2005	weak	moderate	weak	weak	weak	strong	weak	
Fazio et al. 2009	moderate	moderate	weak	weak	weak	strong	weak	
Hecker 1999	weak	moderate	weak	weak	weak	weak	weak	n.a.
Leskow 1985	weak	moderate	moderate	weak	weak	weak	weak	
Marzian 2007	moderate	moderate	weak	weak	weak	weak	weak	
Santoro Júnior 2005	moderate	moderate	moderate	weak	weak	strong	weak	

n.a. = not applicable.

**Table 3:** Summary of the results of included studies.

Reference	Diagnosis	Treatment		Outcomes assessed (selection)*	Results		Statistics**
		Intervention group	Control group		Intervention group	Control group	
Ivy leaf extract versus placebo							

Kemmerich et al. 2006	Acute bronchitis	Ivy + Thyme (syrup)	Placebo	Change in frequency of coughing fits (relative reduction), time to cessation of fits, Bronchitis severity score (BSS), Response to treatment (AD)	Frequency of coughing fits ↓ 77.6%,	Frequency of coughing fits ↓ 55.9%,	*P* < .0001
					Time to a 50% reduction in fits 6d,	Time to a 50% reduction in fits 8d,	*P* < .0001
					BSS ↓ (8.2 →1.6),	BSS ↓ (8.3 →3.3),	*P* < .0001
					Response rate 96.2%	Response rate 74.7%	*P* < .0001
							

Ivy leaf extract versus conventional expectorant therapy							

Bolbot et al. 2004	Acute bronchitis	Ivy (syrup)	Acetylcysteine	FVC, FEV1, PEF, Change in symptoms cough frequency, sputum, shortness of breath, respiratory pain, Global assessment of efficacy (based on symptoms) (AD)	FVC ↑	FVC ↑	s
					FEV1 ↑	FEV1 ↑	s
					PEF ↑	PEF ↑	s
					Global efficacy rated as very good in 40.0%	Global efficacy rated as very good in 12.5%	s
							
							

Comparison of different formulations of ivy							

Unkauf and Friedrich 2000	Acute bronchitis	Ivy (syrup)	Ivy (syrup)	Severity of bronchitis assessed on visual analogue scale (AD), Frequency and quality of cough	Severity ↓ (on scale ↓ 67.3 mm)	Severity ↓ (on scale ↓ 64.2 mm)	*P* = .0031 for equivalence
					After treatment 26.9% without bronchitis	After treatment 36.5% without bronchitis	ns

Observational studies (OSs)							

Büechi and Kähler 2003	Acute bronchitis, common cold	Ivy (pastilles)	n.a.	Change in symptoms urge to cough and sputum quantity on a scale (1–4) (AD), Global assessment of efficacy (AD, AP)	Urge to cough ↓ (2.7 → 1.3)	n.a.	no statistics
					Sputum quantity ↓ (1.5 →1.1)		
					Efficacy rated as very good or good in 78% (AD) and 77% (AP)		
							
Büechi et al. 2005	Acute and chronic bronchitis, common cold	Ivy + Thyme (syrup)	n.a.	Change in symptoms urge to cough, sputum quantity, sputum consistency, ease of expectoration on a scale (1–4), Global assessment of efficacy (AD, AP)	Change of median symptom score for urge to cough, sputum consistency and ease of expectoration 3 → 1, for sputum quantity 2 → 1, Efficacy rated as very good or good in 86% (AD) and 90% (AP)	n.a.	no statistics
Fazio et al. 2009	Acute and chronic bronchitis, cough	Ivy (syrup)	n.a.	Change in symptoms cough, expectoration, shortness of breath, respiratory pain (AD)	Improvement or cure for cough in 93.4%, expectoration 92.9%, shortness of breath 91.2%, pain 90.8%	n.a.	no statistics
Hecker 1999	Bronchitis	Ivy (syrup)	n.a.	Change in symptoms cough, expectoration, shortness of breath, respiratory pain on a scale (1–4) (AD), Global assessment of efficacy (AD)	Improvement or cure for cough in 91.3%, expectoration 87.5%, shortness of breath 57.7%, pain 60.9%, Efficacy rated as very good or good in 86%	n.a.	no statistics
Leskow 1985	Acute and chronic bronchitis, chesty cough, pertussis	Ivy (drops)	n.a.	Change in symptoms cough, expectoration, shortness of breath (AD)	Improvement or cure for cough in 96.4%, expectoration 100%, shortness of breath 100%	n.a.	no statistics
Marzian 2007	Acute bronchitis	Ivy+Thyme (syrup)	n.a.	Change in frequency of coughing fits, Bronchitis severity score (BSS),Improvement yes/no (AD)	Coughing fits ↓ (25.1/d → 4.7/d), BSS ↓ (8.9 → 1.2), Improvement or cure in 94%	n.a.	no statistics
							
Santoro Júnior 2005	Bronchitis	Ivy (syrup)	n.a.	Change in symptoms cough and shortness of breath, Auscultation results (AD)	Cough ↓ (98.8% → 5.4% of patients), shortness of breath ↓ Auscultation: rales ↓	n.a.	s

n.a. = not applicable, ns = not statistically significant, s = statistically significant, FVC = forced vital capacity, PEF = peak expiratory flow, FEV1 = forced expiratory volume in one second, AD = assessment by doctor, AP = assessment by patient, *only outcome parameters reported in a manner allowing comparison between groups are listed (RCT/CCT), for OS: selected relevant outcomes. **if reported: *P*-value for intervention compared to control. For OS: *P*-value for baseline compared to after treatment.
